# Single pediatric center experience with single-use duodenoscopes

**DOI:** 10.3389/fped.2026.1721719

**Published:** 2026-03-31

**Authors:** Sapna Khemka, Micah Morris, Colin Winkie, Muhammad A. Khan

**Affiliations:** Division of Gastroenterology, Hepatology & Nutrition, Nationwide Children's Hospital, Columbus, Ohio, United States

**Keywords:** ASGE ERCP complexity, multidrug resistant organisms, pediatric endoscopic retrograde cholangiopancreatography, post-ERCP complications, single-use duodenoscope

## Abstract

**Background:**

Endoscopic retrograde cholangiopancreatography (ERCP) is a key diagnostic and therapeutic tool for hepato-pancreato-biliary conditions. Although widely performed in adults, ERCP in pediatric populations is less common and poses unique challenges, particularly concerning infection control. Reusable duodenoscopes have been linked to patient-to-patient transmission of multidrug-resistant organisms (MDROs), leading to increased interest in single-use duodenoscopes. This study describes the initial experience using single-use duodenoscopes in ERCP at a single pediatric quaternary care center.

**Methods:**

A retrospective chart review was conducted on all patients who underwent ERCP with the EXALT™ Model D single-use duodenoscope at a single pediatric center from November 1, 2022, to April 15, 2025. Demographics, procedural details, ERCP complexity scores, and post-procedure complications were collected. Descriptive statistics were used for analysis.

**Results:**

Twenty-eight patients (mean age 16 years; 64% female) underwent ERCP with a single-use duodenoscope. Biliary cannulation was successful in 100% of cases. Most procedures were ASGE grade 1 or 2. Stone extraction was performed in 27 cases, stent removal in 25, and new stent placement in 3. Complications occurred in 4 patients (14%): abdominal pain (*n* = 2), post-ERCP pancreatitis (*n* = 1), and cholangitis (*n* = 1), with no cases of post-procedure bleeding or MDRO infections. No procedures required conversion to reusable duodenoscopes.

**Conclusions:**

Single-use duodenoscopes were effective and safe for ERCP in a pediatric cohort, achieving high technical success and low complication rates. While more studies are needed, particularly in younger or smaller patients and at higher ASGE grades (3 and 4), single-use duodenoscopes may offer a promising alternative to reusable devices, especially in reducing infection risk and simplifying scope maintenance. Broader adoption may depend on the development of pediatric-specific devices and cost-benefit analyses in varying clinical settings.

## Introduction

Endoscopic retrograde cholangiopancreatography (ERCP) is a widely used diagnostic and therapeutic intervention for several hepato-pancreato-biliary conditions, the most common indication being choledocholithiasis (1). Pediatric patients represent a minority of total ERCPs performed, with one study finding 11,060 hospitalized pediatric (age <20 years) patients who underwent ERCP between 2005 and 2014 in the National Inpatient Sample database (2).

Reusable duodenoscopes, used for ERCP procedures, have been associated with patient-to-patient duodenoscope related infections, with recognition in the past decade of multiple outbreaks with multidrug-resistant organisms (MDRO) (3). A recent FDA surveillance study showed a duodenoscope contamination rate of 9%, with 5.4% caused by high-concern organisms (4). A metanalysis recently reported a 15.25% contamination rate of patient-ready processed duodenoscopes (5). There have been cases of multidrug-resistant organisms transferred to patients after procedures with reusable duodenoscopes, with infections caused by organisms such as carbapenem-resistant *Escherichia coli*, carbapenem-resistant *Enterobacteriaceae*, and most commonly *Klebsiella pneumoniae* and *Pseudomonas aeruginosa* (6–8). More aggressive reprocessing strategies have been recommended by the FDA to help address these outbreaks (4). Additionally, to circumvent this risk associated with reusable duodenoscopes, single-use duodenoscopes have been developed and approved by the FDA for use, with first approval in December 2019 for the EXALT™ Model D (Boston Scientific Corporation, Marlborough, MA) followed by the aScope™ Duodeno (Ambu Inc., Columbia, MD) in 2020. At our center, single-use duodenoscopes have been used in select cases since 2022. This retrospective review aims to describe experience with single-use duodenoscopes at a single quaternary pediatric center in a cohort of patients.

## Materials & methods

The study was approved by Nationwide Children's Hospital Institutional Review Board (Study 4898).

Data was collected through retrospective chart review of patients who had procedures performed at our center utilizing an EXALT Model D single-use duodenoscope. Data reviewed was stored by Epic Electronic Medical Records (Epic Systems Corporation, Verona, WI) and endoscopy documentation software ProVation® MD (Provation Software, Inc., Minneapolis, MN). Patient demographics at time of procedure, including sex, age, and body mass index, were documented. History of previous ERCP and biliary sphincterotomy, indications for the procedure, procedure success or failure, total procedure time, American Society for Gastrointestinal Endoscopy (ASGE) ERCP complexity grade, need to crossover to a reusable scope, cannulation of the papilla, and specific details of the procedures including if sphincterotomy, sphincteroplasty, stone extraction, stent removal, or stent placement were performed was also documented. Complications including pain, pancreatitis, cholangitis, and bleeding within 30 days following the procedure were identified on chart review. Pain was defined by patient requiring increased pain medications post-ERCP compared to prior with an associated increase length of hospital admission. Post-ERCP pancreatitis was also characterized by new or worsening pain combined with elevated serum pancreatic enzymes (amylase and/or lipase) requiring hospitalization or prolonging admission. Cholangitis was suspected if the patient developed fever and worsening transaminases on lab evaluation, as defined by the primary team. Bleeding was identified by physical signs, including hematemesis, hematochezia, or melena, associated with vital sign changes and declining hemoglobin. Data was stored in a de-identified database in Microsoft® Excel (Microsoft Corporation, Redmond, WA) for analysis with descriptive statistics.

## Results

This study included 28 patients who underwent ERCP using a single-use duodenoscope from November 1, 2022 to April 15, 2025 at a quaternary pediatric center. All procedures were performed by the single interventional endoscopist at our center. The patients ranged from 11 years old to 24 years old, an average of 16 years old. Eighteen of the patients were female. The average weight of all patients was 73.5 kilograms and average body mass index (BMI) 27.3. The total procedure time ranged from 14 min to 1 h and 59 min, the average being 32 min. Patient demographics and procedure times are summarized in Table 1.

**Table 1:** Summary of patient demographics, procedure details, and post-operation complications.

Patient and procedure characteristics	Patients (*n* = 28)
Patient Demographics	
Age, mean (range)	16.2 years old (10.6–24.3)
Female (%)	18/28 (64%)
Weight, mean (range)	73.5 kg (33.3–150.2)
BMI, mean (range)	27.3 (16.35–55.16)
Total Procedure Time, mean	32 min
Cannulation	
Not intended	-
Straight	27/28 (96%)
After Adjunct	1/28 (4%)
Unsuccessful	-
Previous ERCP	27/28 (96%)
Biliary Sphincterotomy	
Previous	26/28 (93%)
*de novo*	1/28 (4%)
No Sphincterotomy	1/28 (4%)
Sphincteroplasty	1/28 (4%)
Stone Extraction	27/28 (96%)
New Stent Placement	3/28 (11%)
Stent Removal	25/28 (89%)
Post-Operation Complications	
Pain	2/28 (7%)
Pancreatitis	1/28 (4%)
Cholangitis	1/28 (4%)
Bleeding	-
Concomitant Laparoscopic Cholecystectomy	10/28 (36%)

Cannulation into the ampulla and subsequently into the bile duct was intentional and successful in all patients, straight cannulation in 27 patients and after adjunct therapy in one patient who required a double wire technique. Most of the patients had undergone previous ERCP and biliary sphincterotomy prior to ERCP with single-use duodenoscope. Only one of the 28 patients underwent *de novo* biliary sphincterotomy and sphincteroplasty. The procedure for all patients included a balloon sweep of the biliary duct that resulted in stone extraction in 27 cases, stent removal in 25, and a new stent placement in 3. Out of all patients, 4 experienced complications. This included pain in 2 children, post-ERCP pancreatitis in 1 child, and cholangitis in 1 child. No patients experienced bleeding or infection with MDRO post-operatively. Concomitant laparoscopic cholecystectomy was performed in 10 patients at the time of ERCP. These results are summarized in Table 1.

**Figure 1:**
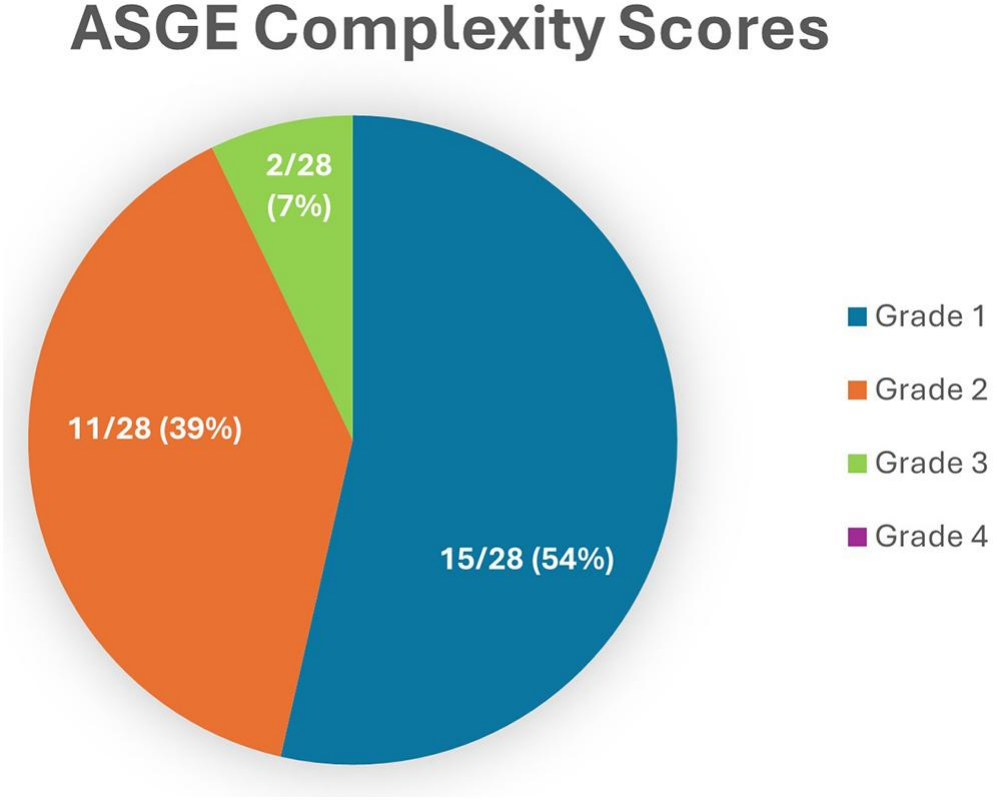
Breakdown of ASGE complexity grades for all procedures performed with a single-use duodenoscope.

The ASGE developed a grading system for assessing the difficulty of an ERCP, ranging from grades 1–4 with 4 signifying the most complex ERCP procedure often requiring advanced techniques. As outlined in Figure 1, most procedures were ASGE 1 and 2. No patient had a complexity score of 4.

## Discussion

In this study, we retrospectively evaluated the use of single-use duodenoscopes in ERCP at a single pediatric center by analyzing endoscopic techniques performed and clinical outcomes in 28 patients. The results demonstrate that single-use duodenoscopes can be successfully used to cannulate the bile duct in 100% of patients, similar to rates of targeted duct cannulation in over 95% of standard pediatric ERCP cases (9, 10). Previous pediatric ERCP experience supports higher success in procedures classified as ASGE grades 1 and 2 compared to grades 3 and 4, supporting the successful technical rates seen in this study as most patients met criteria for ASGE grades 1 and 2 (10).

The single-use duodenoscope can also be used to perform common therapeutic interventions including sphincterotomy, stone extraction, and stent placement or removal with minimal complication. A multicenter prospective collaborative study assessing standard pediatric ERCPs demonstrated that overall adverse events related to pediatric ERCPs are uncommon, occurring in less than 10% of almost 1000 patients analyzed (10). These rates of adverse events are similar in adult single-use duodenoscope procedures (11). Our series shows similarly low ERCP-related complication rates in this pediatric cohort when utilizing a single-use duodenoscope.

Patients who have previously undergone ERCP and have a history of antibiotic use are known to have MDRO presence (12). Utilization of a single-use duodenoscope may decrease nosocomial infections by avoiding a possible MDRO infection inoculated in reusable scopes while also decreasing costs associated with reprocessing and maintaining reusable duodenoscopes (13). There were no complications of MDRO infection in this case series, although theoretical detection of an infection would be unusual in this small cohort as its prevalence is very low (14).

Centers where ERCPs are performed should also evaluate the financial implications of reusable vs. single-use equipment. Reusable duodenoscopes, while expensive upfront, come with maintenance and repair costs annually with additional reprocessing labor costs (15). Calculated costs are higher when factoring infection rates and treatment costs of nosocomial infections (15). Singe use duodenoscopes provide an alternative financial model that could offset some of the additional costs incurred by reusable equipment, especially at low volume ERCP centers.

Limitations of this study include the retrospective design, being unable to randomize patients in a prospective manner. The small sample size of patients that underwent ERCP with single-use duodenoscope poses as a limitation as well. This may affect the evaluation of complication rates present. Additionally, most patients had previously undergone an ERCP limiting the evaluation of first-time success rates and complications. High cannulation rates also likely reflect high proportion of patients undergoing repeat ERCP and low ASGE complexity (grades 1 and 2). Patient demographics were older children and adolescents, most of whom were adult size. Finally, all procedures analyzed were performed at a single center by the only interventional endoscopist limiting the variety of resources used and techniques applied.

A multicenter study with a larger sample size would be of benefit to further evaluate if there is bias towards ERCP procedures in older and larger sized pediatric patients as demonstrated by this current study. Furthermore, analyzing how single-use duodenoscope success and complications rates would vary in patients with non-native papillary cannulation may be helpful to assess a more general utility of the equipment. Future studies may also elicit the need for more availability of pediatric sized single-use duodenoscopes, specifically for younger aged children and smaller sized patients.

The challenge remains in the use of duodenoscopes in smaller children. There is an overall lack of ERCP equipment presently available on the market to help care for this patient population. Solutions geared towards reducing infection rates in reusable duodenoscopes with disposable end-caps have shown greater challenges in children, specifically finding higher than anticipated rates of mucosal trauma, post-ERCP pancreatitis, and difficulty with cannulation (16). The development of small caliber single-use duodenoscopes would expand the therapeutic options available to children needing ERCP (17). There would be great opportunity to develop a single-use duodenoscope for children, especially given the simpler manufacturing techniques needed.

## Data Availability

The raw data supporting the conclusions of this article will be made available by the authors, without undue reservation.
